# Design and Control for WLR-3P: A Hydraulic Wheel-Legged Robot

**DOI:** 10.34133/cbsystems.0025

**Published:** 2023-06-08

**Authors:** Xu Li, Haoyang Yu, Haibo Feng, Songyuan Zhang, Yili Fu

**Affiliations:** ^1^ State Key Laboratory of Robotics and System, College of Mechatronics Engineering, Harbin Institute of Technology, Harbin 150001, China.; ^2^ State Key Laboratory of Fluid Power and Mechatronic Systems, Zhejiang University, Hangzhou 310027, China.; ^3^ Intelligent Robot Research Center, Zhejiang Laboratory, Hangzhou 311100, China.

## Abstract

The robot used for disaster rescue or field exploration requires the ability of fast moving on flat road and adaptability on complex terrain. The hybrid wheel-legged robot (WLR-3P, prototype of the third-generation hydraulic wheel-legged robot) has the characteristics of fast and efficient mobility on flat surfaces and high environmental adaptability on rough terrains. In this paper, 3 design requirements are proposed to improve the mobility and environmental adaptability of the robot. To meet these 3 requirements, 2 design principles for each requirement are put forward. First, for light weight and low inertia with high stiffness, 3-dimensional printing technology and lightweight material are adopted. Second, the integrated hydraulically driven unit is used for high power density and fast response actuation. Third, the micro-hydraulic power unit achieves power autonomy, adopting the hoseless design to strengthen the reliability of the hydraulic system. What is more, the control system including hierarchical distributed electrical system and control strategy is presented. The mobility and adaptability of WLR-3P are demonstrated with a series of experiments. Finally, the robot can achieve a speed of 13.6 km/h and a jumping height of 0.2 m.

## Introduction

### Background

**Fig. 1.**
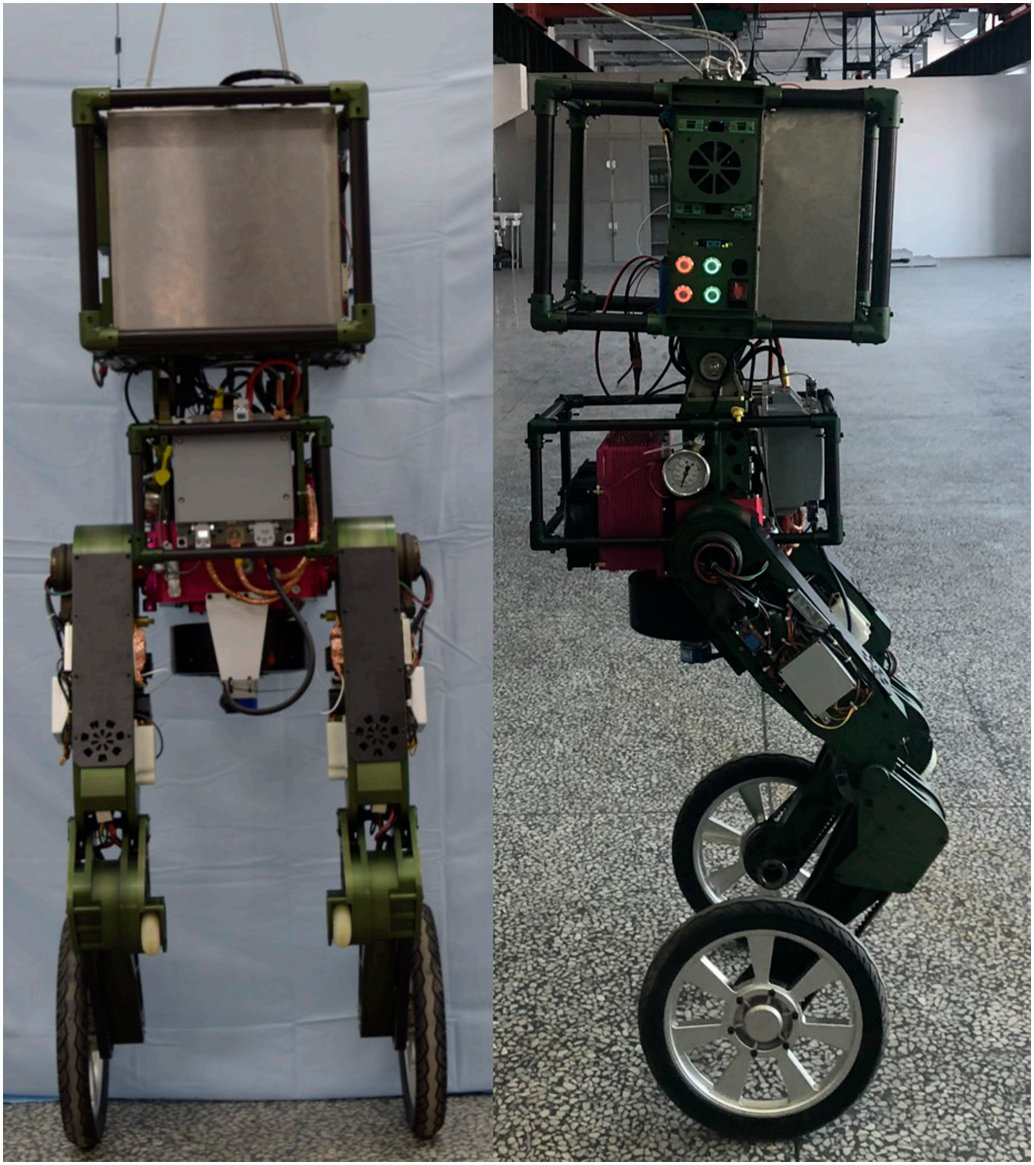
WLR-3P, a novel hoseless hydraulic wheel-legged robot that is capable of jumping and fast-moving with the autonomous power.

As highlighted in 2015 DARPA Robotics Challenge [1], exploration and rescue in dangerous environment are important applications for robotic legged locomotion, which remain unsolved, however. Dynamic legged robots are superior to wheeled vehicles in terms of mobility on rough terrain. During the past decades, many bipedal robots [2–5] have been developed with improved abilities of balancing, push recovery and running, as well as capability of moving on irregular terrain. Compared with wheeled and legged locomotion, the multifunction, maneuverability, and speed of dynamically stabilized wheel-legged machines in continuous and smooth terrain are still the preferred platforms. Leg provides better mobility in rough terrain, and wheel realizes fast movement over a continuous path. Consequently, the combination of wheels and legs enables the robot to adapt to almost all terrains. In the DRC [DARPA (Defense Advanced Research Projects Agency) Robot Challenge] finals, excellent results have been achieved by the wheel-legged robots, and 4 of the 5 best-placed teams chose to combine leg with wheeled locomotion, which might indicate that this design approach has advantages in dealing with challenging tasks [6–9]. As shown in Fig. 1, this paper systematically introduces the development process and experimental results of WLR-3P, which is a typical wheel leg compound motion robot by adding wheels at the end of the legs.

### Why could hydraulics be a good choice

Before describing the details, we will briefly discuss why we chose hydraulics as major driving mode. We believe that the hydraulic actuation is suitable for legged robot platforms considering the following reasons: (a) high power-to-weight ratio, (b) excellent impact resistance, (c) high-frequency response of force/position control, and (d) human-comparable biological properties.

Among the reasons above, items (a) and (b) are the main reasons. The maximum power density of the knee joint hydraulically driven unit (HDU) in WLR-3P can reach about 7 kW/kg, which is far higher than that of traditional motors. Due to the fluidity of the oil, the hydraulic drive system should be able to share the hydraulic power output from the HPU. It is easy to concentrate the hydraulic power to a certain HDU output so that it can achieve high power. It is difficult for electrohydrostatic actuation to generate instantaneous high power because of the limited power of the motor [10]. Shock resistance and overload protection are required by robots that work with humans or carry out rescue activities in disaster areas. Fluidic actuations like hydraulics or pneumatics are of strong shock resistance due to their intrinsic compliance.

Item (c) should be the most attractive point for most legged robot researchers. Hydraulic actuation ensures high enough actuator bandwidth to achieve satisfactory force/displacement control in the spectrum of high dynamic locomotion of legged robots [11]. The force controllability, which is the basis of efficient compliance behavior through natural and stiff actuation system, can be extended further by using advanced model-based control methods [12]. Existing actuations meeting the stringent requirements (fast force response and precise position tracking actuation with high impact robustness) also includes the proprioceptive actuation [13] and the series elastic actuation (SEA) [14]. The proprioceptive actuation implemented on MIT Cheetah [15] can perform tasks like high-speed running and jumping. Unfortunately, the power density of electric motors is limited. Several state-of-the-art robots like the humanoid Valkyrie [16] or the quadruped Anymal [17] showed how to use SEA not only for precise output force regulation but also for storing energy temporarily during locomotion. However, SEA-equipped robots suffer from severe latency and limited bandwidth. Additionally, in posture measurement of robots, high-speed locomotion will also produce severe vibration and noise pollution [18].

Item (d) is considered as very suitable for the combination of humanoid robots. Mechanically robust cylinders with single rod are very compact, and if the linkages are properly designed, they can be well suited to driving the robotic locomotion and jumping. The robot can readily obtain different torques and speeds for different joints merely by changing the piston diameter size and the valve type [19]. Furthermore, the hydraulic fluid can also serve as the lubricant and coolant of actuators. The flow characteristics of hydraulic oil similar to human blood can transfer local heat to the whole body of the robot, as well as transmits power to each HDU, which is beneficial to the heat dissipation.

Nevertheless, there are several shortcomings of a hydraulic system, such as leakage and radiate heat. Therefore, cooling is particularly important when the servo valve-driven systems run for a long time. How to transfer the hydraulic power of HPU to each actuator is another concerning problem. In the structure of the robot, the hydraulic power transmission is realized by the hoseless hydraulic joints with flow path sealed by a rotary seal and integrated design with cylinder, servo valve, and skeleton [20]. This is also similar to the organic fusion of human blood vessels and musculoskeletal. The hoseless design method integrated with the structure is helpful to improve the heat dissipation efficiency, and the hydraulic power transmission efficiency is increased also because of the shorter oil pipeline. The integrated design reduces the sealing and the possibility of leakage. Hyon et al. [19] used external hydraulic power supply and rubber hoses to realize hydraulic power transmission between HPU and HDUs. However, power autonomy is an essential condition for robots to realize independent outdoor movement. It is very difficult for a robot to integrate HPU, including miniaturization of pump, motor or engine, and integration of various hydraulic components. The solution proposed in this paper can solve this problem well. Only with strong driving ability and powerful actuation, the robot can obtain high dynamic motion ability. We believe that hydraulic actuation is one of the best drives for a legged robot in the future.

### Related work

Inspired by Momaro [8], CENTAURO [21] as a wheel-legged mobile manipulation platform can execute demanding manipulation tasks and demonstrate substantial physical resilience. Ascento [22] is a compact wheeled bipedal robot that is able to move quickly on flat terrain and to overcome obstacles by jumping. Handle [23] is also a biped robot—but with wheels instead of feet, which combines the rough-terrain capability of legs with the efficiency of wheels. Nevertheless, so far, there have been no published papers concerning Boston Dynamics’ locomotion framework and detailed designs of Handle. Through the above motion characteristics of these robots, we can get that the driving wheel is designed to replace the foot at the end of the leg, which greatly improves the movement ability of the robots. Due to its small footprint, bipedal wheel-legged robots have been more extensively studied in recent years. Table 1 shows the main parameters of several wheel-legged robots. It is not difficult to find that hydraulically driven wheel-legged robots have obvious advantages in terms of load capacity and explosive power.

**Table 1.** Comparison of parameters of several wheel-legged robots.

Robot	Department	Actuation	Weight (kg)	Height (m)	Maximum load (kg)	Maximum velocity (m/s)	Jump height (m)
Handle [23]	Boston Dynamic	Hydraulic	90	1.98	30	14.18	1.22
WLR-3P	HIT	Hydraulic	80	1.55	35	13.6	0.2
SDU-WLR [32]	SDU	Hydraulic	80	1.25	Unknown	3.6	-
Ascento [22]	ETH	Motor	10.4	0.66	Unknown	8	0.1
SR600 [33]	HITsz	Motor	14	0.6	Unknown	7.2	-
Ollie [34]	Tencent X	Motor	11.3	0.916	12	Unknown	-
LGOR [35]	HEBI	Motor	17.5	1	Unknown	3.6	-
Diablo	Direct drive tech	Motor	22.9	0.49	4	7.2	0.14

**Fig. 2.**
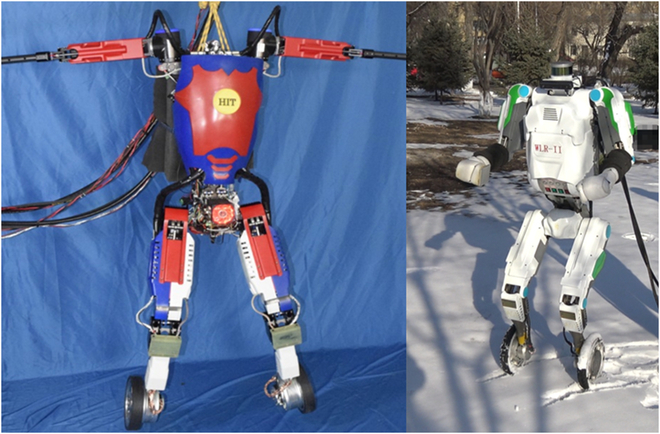
Left: WLR-I, the first-generation hydraulic wheel-legged robot, which was in joint motion test. Right: WLR-II, the second-generation hydraulic wheel-legged robot, which was on snowfield moving test.

In 2017, we developed the first hydraulic wheel-legged robot WLR-I [24], which successfully verified the integrated design and compound movement ability. Then, in 2018, we developed the second-generation wheel-legged robot (WLR-II [20]), which realized hoseless design for the first time and showed unexpected terrain adaptability. As shown in Fig. 2, the first 2 generations have verified the adaptability to complex terrain and the mobility on flat ground, but both are powered by the external pump station. Based on the design of the previous 2 generations of wheel-legged robots, substantial improvements have been made in WLR-3P. WLR-3P not only improves the motion ability but also realizes the power autonomy.

### Contributions

The main contributions of this paper are as follows:

1. A hydraulic power autonomous wheel-legged robot system with high mobility and high adaptability is developed. It is very difficult to integrate hydraulic power system inside the robot, especially in the very narrow space of the bipedal wheel-legged robot. The WLR-3P robot developed in this paper has successfully integrated HPU and achieved rapid movement and jumping motion, which provides substantial verification for the research and development of bipedal wheel-legged robot.

2. Illustrates of the solution procedures for these issues with basic templates such as actuator and transmission characterization, sensor design for impact, and leg morphology for a real machine. The hydraulically driven robot is a minority in the field of robotics, but its powerful explosive force and impact resistance make it an excellent choice for high-performance motion robots. However, few papers report the detailed design methods and specific parameters of the drive system and structure of the hydraulically driven robot. In this paper, the design methods and results of hydraulic bipedal wheel-legged robot are summarized from the experience of developing these hydraulically driven robots, which provide a detailed technical reference for peers.

3. A novel hoseless design and a good technical guideline for building superior hydraulic wheel-legged robots were proposed. One of the most difficult technologies in the development of hydraulically driven robot is the integration design of complex hydraulic system and mechanical body. In this paper, the hoseless design method of hydraulically driven robot is proposed. The hydraulic system and mechanical body are integrated through the oil joint, airborne HPU, and integrated HDUs, which improves the reliability and compactness of the system.

To the authors’ best knowledge, there is no relevant literature that fully covers these topics. The paper is organized as follows. The “Overview of the Novel Wheel-Legged Robot: WLR-3P” section addresses the characteristics and composition of WLR-3P. The design principles and their implementation are introduced in the “Design Principles and Implementation” section. The “Control System of WLR-3P” section shows the control system of WLR-3P. The “Experimental Results” section shows serial experimental setups and results. Conclusions and future works are given in the “Conclusions” section.

## Overview of the Novel Wheel-Legged Robot: WLR-3P

**Fig. 3.**
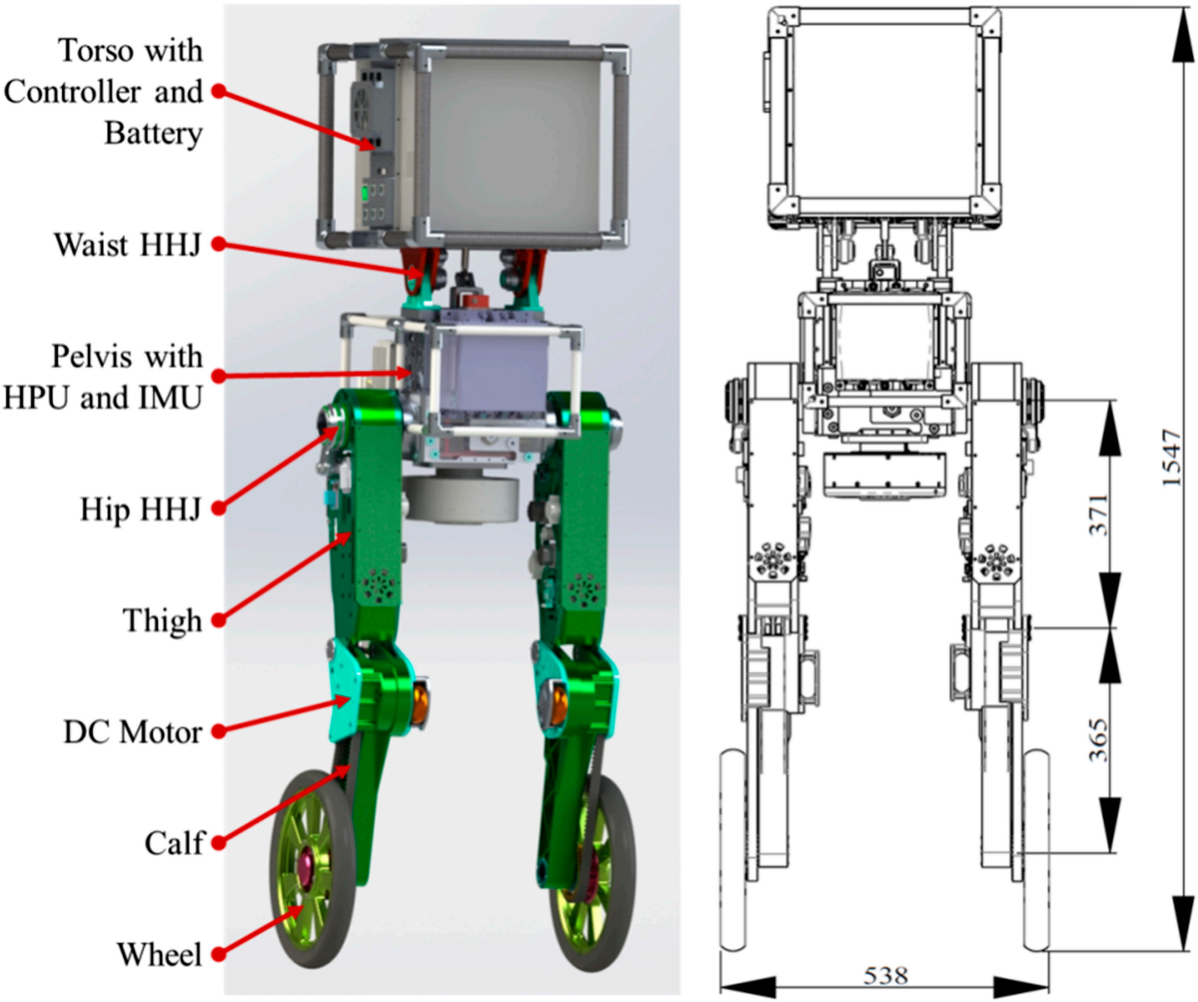
Component composition and dimensions of WLR-3P.

This section mainly introduces the overview of WLR-3P, as shown in Fig. 3. WLR-3P has 7 degrees of freedom (DOFs): 3 DOFs in each leg and 1 DOF in the waist. The 3 DOFs of the leg are respectively on the hip joint, knee joint, and driving wheel, in which the hip and knee are driven by HDU and the driving wheel is driven by DC motor. HDU includes the customized high-frequency response (20 Hz) servo cylinder, a high-performance servo valve, a displacement sensor, and a force sensor. WLR-3P with an airborne HPU and batteries weighs 80 kg. Its maximum height is 1.55 m, and the distance between the 2 wheels is about 0.54 m. Additionally, a 1.8-kg carbon fiber mixed with aluminum alloy frame is attached to the trunk for safety.

WLR-3P is a wheel-legged robotic platform that can be used for high-speed motion, dynamic balance on the irregular ground, and jump over obstacles. To obtain the flexibility for accomplishing different tasks, WLR-3P is designed with flexible mechanical properties and adequate controllers. The main specifications of WLR-3P are presented in Table 2.

**Table 2.** Main specifications of WLR-3P.

Mass	80 kg
Size	Height, 1.55 m; width, 0.54 m
DOFs	2 × leg (3), waist (1)
Actuation	Hydraulic cylinder
Power supply	7.5 kW HPU (internal)Pressure, 12 MPa (normal), 21 MPa (max)72 V lithium battery, 30 Ah
Controllers	32-bit QNX on PC/104 (onboard) Intel Atom D525 (1.8 GHz, Dual-core)
Actuator sensor	LVDT, load cell, pressure sensor, incremental encoder

## Design Principles and Implementation

**Fig. 4.**
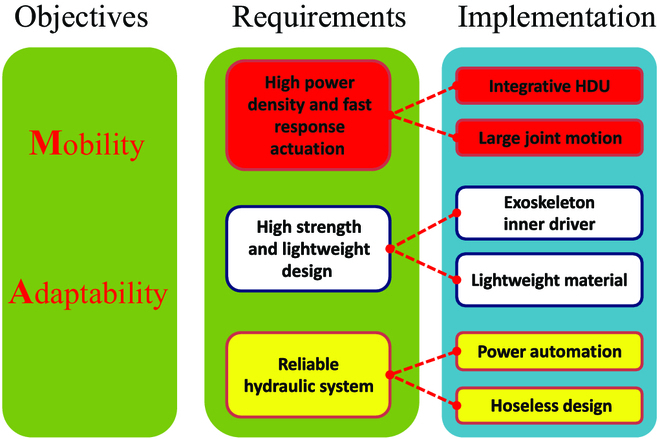
Mechanical design method for WLR-3P.

In this section, the design principles and their implementation of WLR-3P are introduced, as shown in Fig. 4. WLR-3P is characterized by mobility on flat ground and strong adaptability on complex terrain. Based on these 2 objectives, this work proposed 3 requirements for designing WLR-3P: (a) high power density and fast response actuation, (b) light weight and inertia with high strength, and (c) reliable hydraulic system.

### High power density and fast response actuation

#### Integrative hydraulic drive unit

In the design of HDU, the relation between cylinder/rod diameter and load, stroke and joint angle, servo valve flow, and joint speed should be analyzed. Combined with (considering) the experience of previous actuation design and the existing standard parts size, a cylinder/rod diameter of 20/12 mm is adopted. The cylinder can exert a push force of 3,770 N and a pull force of 2,413 N. The values increase to 6,597 and 4,222 N, respectively, at 21 MPa (3,000 lbf/in^2^). The stroke of the cylinder is directly related to the range of joint angle. The range of joint angle is also related to the size of the transmission mechanism, which needs to be considered comprehensively in the design process. The joint angular velocity is related to the flow rate of the servo valve. According to the structure and weight requirements, this work directly selects the Star 200 servo valve, which weighs 230 g. Star Type 200 valve offers 7 l/min at 7 MPa pressure drop, with a frequency response of 130 Hz at −3 dB and 90 phase lag for ±25% input. The relationship between load flow *Q_L_* at valve orifice and supply pressure *P_S_* under zero load *P_L_* = 0 can be expressed as $\;Q_{L}=K_{Q}\sqrt{P_{S}}$, where *K_Q_* stands for flow proportional coefficient. The higher the value of *P_S_*, the greater the amount *Q_L_* can flow into the actuator from the high-pressure line, resulting in higher speed. The maximum speed of expansion and contraction of piston can reach 0.643 m/s and 1 m/s, respectively.

Reasonable design parameters should not only meet the requirements of load and movement but also reduce the cost, as well as improve the interchange capacity of standard parts and system maintainability. On top of that, for the design of HDU, the control requirements, displacement/speed feedback, and force feedback are needed in force/position control. Therefore, each HDU integrated with a linear variable differential transformer (LVDT) is parallel to the piston rod for displacement/speed feedback and a load sensor in coaxial series with the piston. To improve the response speed and load stiffness of HDU, the servo valve is installed near the hydraulic cylinder.

#### Transmission design for the joint

The traditional hydraulic robot joint drive uses the expansion and contraction of cylinder and piston rod to directly drive the joint rotation. The joint bearings are used at both ends of the cylinder to compensate the mechanical eccentricity. The design proposed in this paper is to integrate the cylinder with the skeleton and convert the linear motion of the piston rod into the rotation of the joint through the crank slider mechanism. The advantage of this design is that the piston rod only bears the axial force, and the radial force caused by the joint rotation is borne by the linear guide rail, which greatly improves the service life and reliability of the cylinder.

**Fig. 5.**
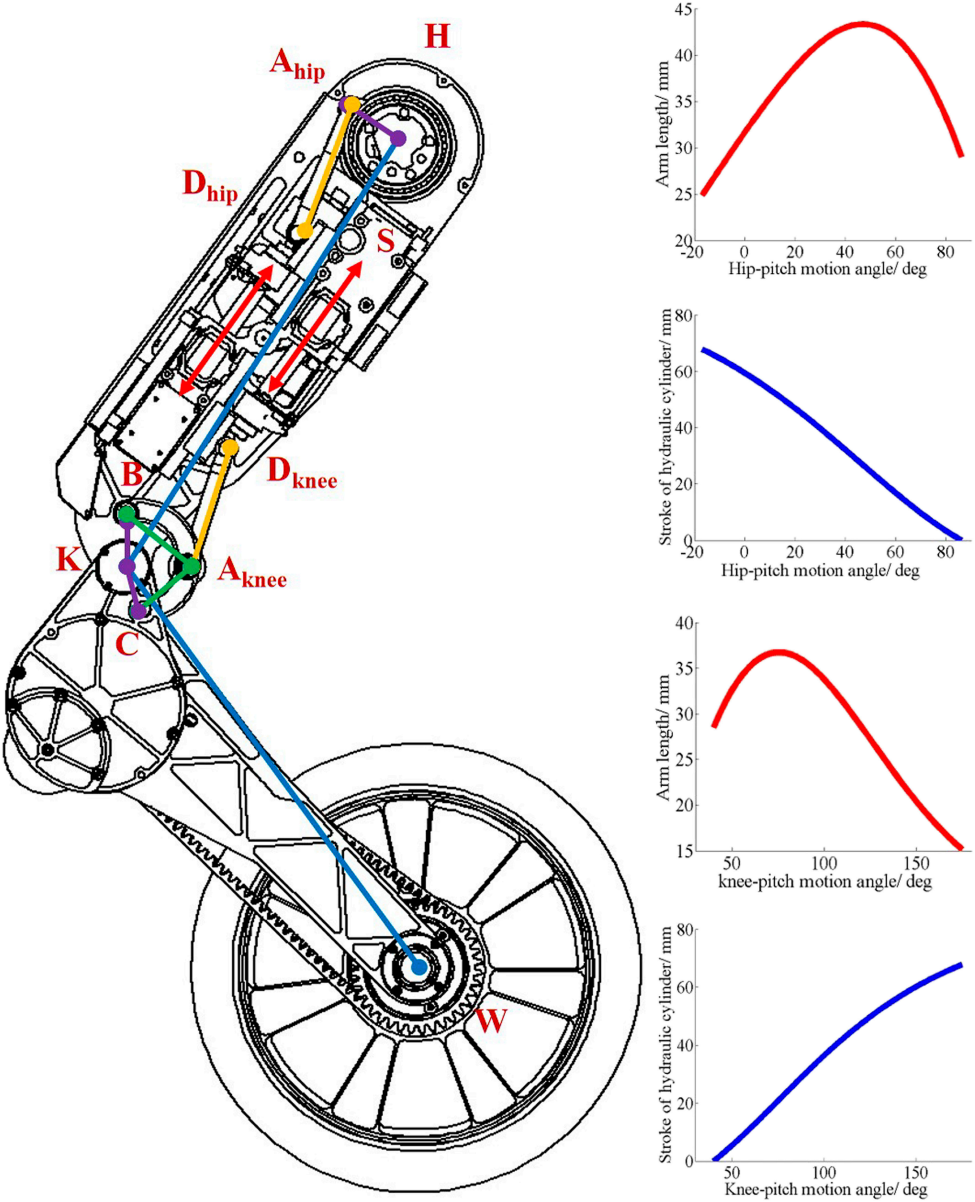
Transmission diagram of knee and hip. The knee joint is dragged by 4-bar linkage, and the hip-pitch joint is dragged by a crank slider mechanism. The small pictures of the red track on the right show the relationship between the moment arm length and the angle of hip-pitch and knee, respectively. The small figures of blue trajectory show the relationship between the joint angle of hip-pitch and knee and the cylinder stroke, respectively, which can be approximately linearized by appropriate parameters of linkage mechanism.

Figure 5 shows the transmission structure for the knee and hip joints adopted in the final design. The piston makes a linear reciprocating motion under the guidance of the linear guide rail. The hip joint rotates under the push of 2-force bars *l*_ADhip_. The squat and jump motion require that the knee has large torque and fast angular velocity. The rotation of the knee is transformed by a 4-bar linkage [25]. Reasonable size of the 4-bar link can realize the large range of knee rotation and the linear conversion of displacement angle. Figure 5 also shows the relationship between joint angle and cylinder displacement and moment arm length. The transmission mode of the waist joint is similar to that of the hip joint.

Table 3 shows the detailed parameters of each joint. The maximum torque of the joint is obtained by the product of the maximum load force of the cylinder and the maximum moment arm length. The maximum load force of cylinder is obtained under the rated pressure of 21 MPa. The maximum angular velocity is obtained from the fastest speed of the cylinder. The torques and velocities are evaluated at the maximum and minimum moment arm, respectively.

**Table 3.** Joint range, angular velocity, and driving torque of WLR-3P.

Joint	Cylinder/rod-stroke (mm)	RoM (deg)	Maximum torque (Nm) @ 21 MPa	Maximum angularvelocity (rad/s)
Knee	20/12-68	39.94–175.64	242/99	27/67
Hip	20/12-68	−16.9 to 86.41	286/165	23/40
Waist	20/14-45	−31.49 to 28.44	297/198	22/33

#### HDU experiments

WLR-3P knee and hip cylinders were taken as experimental objects to verify the performance of HDU in no-load speed, displacement tracking, and force tracking.

**Fig. 6.**
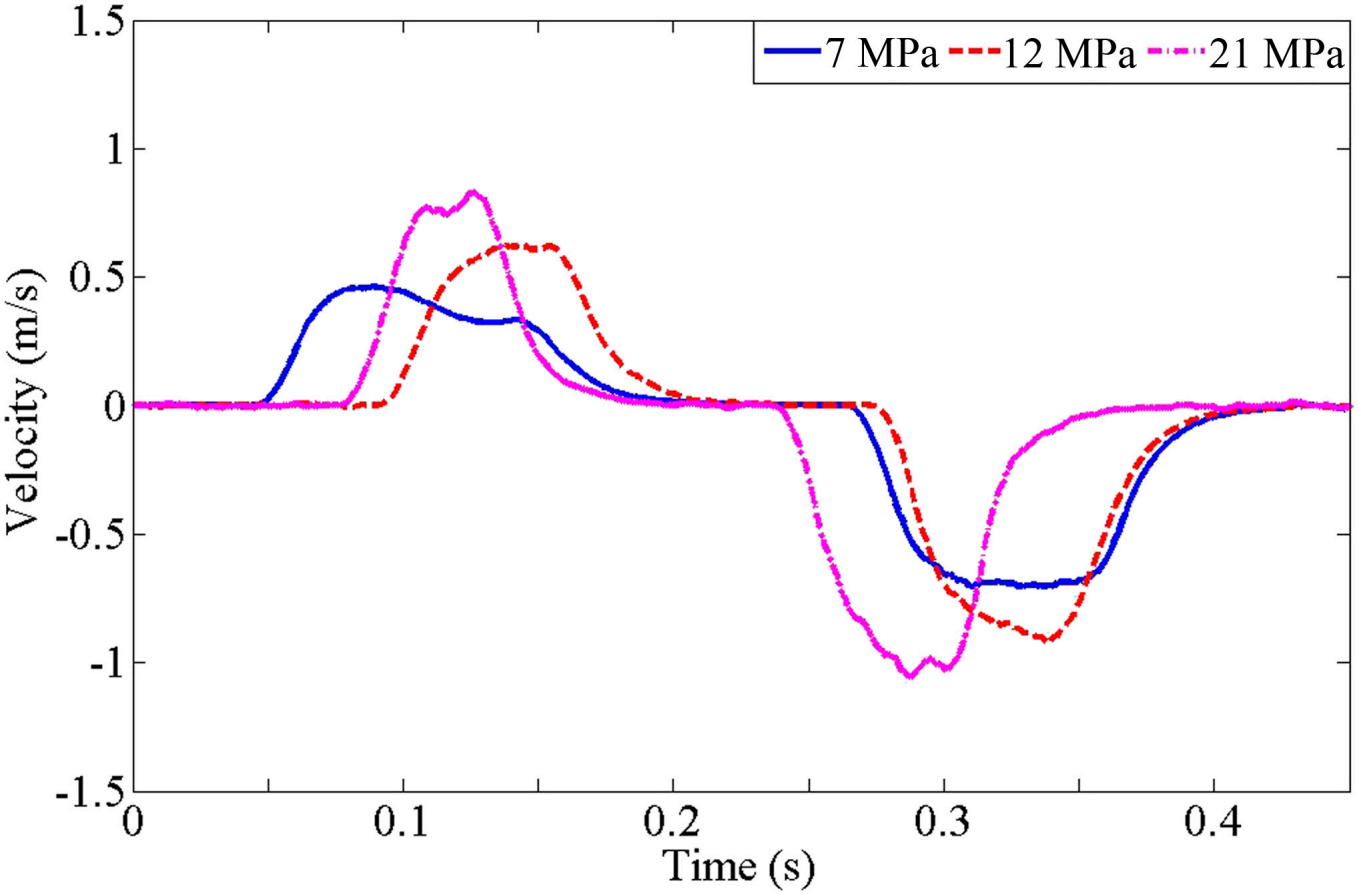
The no-load speed test results at 7 MPa (rating), 12 MPa (normal), and 21 MPa (maximum).

In the no-load speed test, the piston rod can move freely without load. The system supply pressure is set at 7 MPa (rating), 12 MPa (normal), and 21 MPa (maximum). The servo valve signal is switched back and forth between maximum current (+30 mA) and minimum current (−30 mA) at a given interval time. In Fig. 6, the experimental results show that the maximum speed is approximately 0.7 m/s at 21 MPa supply, 0.6 m/s at 12 MPa supply, and 0.45 m/s at 7 MPa supply when the high pressure acts on the rodless cavity. When the high-pressure acts on the rod cavity, the maximum speed is approximately 1.05 m/s at 21 MPa supply, 0.8 m/s at 12 MPa supply, and 0.6 m/s at 7 MPa due to the smaller action area. The difference between the actual and the theoretical value is due to the deviation of the actual flow pressure coefficient of the servo valve and the friction of the servo cylinder.

**Fig. 7.**
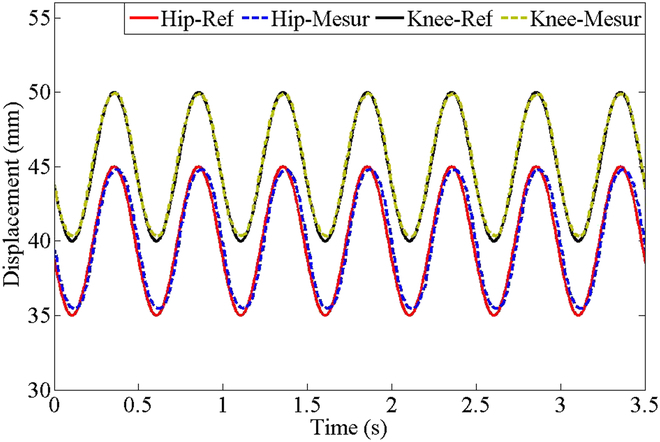
Sinusoidal displacement tracking experiment of hydraulic cylinder for knee and hip joints at 12 MPa supply. Legend items with “-Ref” mean “desired.” Legend items with “-Mesur” mean “Measured.”

Figure 7 presents the experimental result of sinusoidal displacement tracking comprising hip and knee joints. A simple proportional controller is adopted and the supply pressure is set at 12 MPa in the test. The expected hip joint cylinder command is a sinusoidal displacement with an intermediate value of 40 mm, an amplitude of 5 mm, and a frequency of 2 Hz. The expected command of the knee joint cylinder is a sinusoidal displacement with an intermediate value of 45 mm, an amplitude of 5 mm, and a frequency of 2 Hz. When the piston retracts, the tracking error is greater than that when it extends out. This is because the piston is driven by high pressure in the rod cavity during retraction, and the thrust force is smaller under the same pressure. Compared with the hip joint, the knee joint has a smaller tracking error because the knee joint conveys less inertia.

**Fig. 8.**
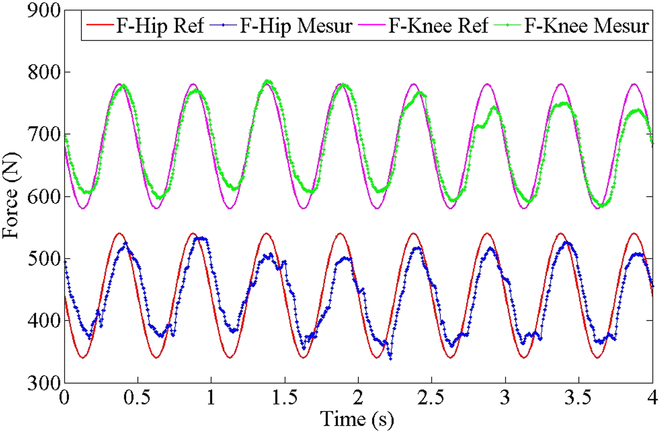
Sinusoidal force tracking experiment of hydraulic cylinder for knee and hip joints at 12 MPa supply.

In the force tracking experiment, this paper adopted a proportional–integral controller because of the noise of the force sensor. In this test, the supply pressure is 12 MPa, and the wheel keeps contact with the ground. Figure 8 presents the experimental result of sinusoidal force tracking comprising hip and knee joints. The median of sinusoidal expected force for hip joint and knee joint is 440 and 680 N, respectively, and the amplitude is 100 N with 2-Hz frequency. The preliminary experimental results show that HDU of WLR-3P can track the expected force command.

### High strength and lightweight design

Light weight and high strength were crucial criteria for the WLR-3P design not only to achieve higher jumps but also to minimize impact forces during landing and takeoff.

#### Thigh design

**Fig. 9.**
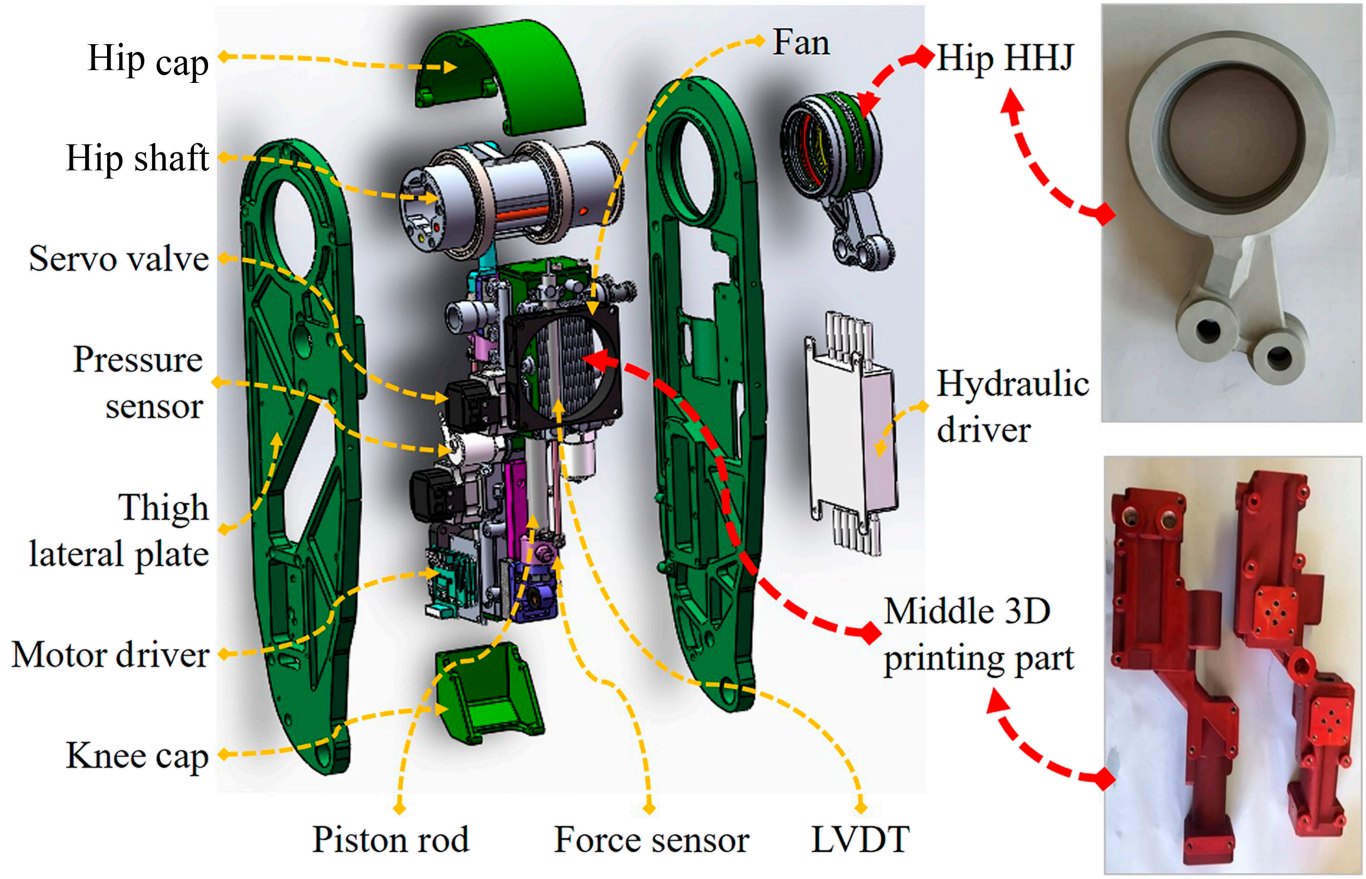
The structure and composition of the thigh.

The structure and composition of the thigh are shown in Fig. 9. The concept of thigh design is to integrate hydraulic cylinders and complex pipes into the middle 3-dimensional (3D) printing part by additive manufacturing and to process the main load-bearing skeleton from hip to knee through subtractive manufacturing. The thigh lateral plate and the middle printing part are fixed by screws. The thigh integrates hydraulic cylinders, pistons, servo valves, LVDTs, force and pressure sensors, etc. What is more, the 2-channel hydraulic driver and the motor driver of the driving wheel on the calf are also installed on the side of the thigh. The hip cap and knee cap, attached to the thigh lateral plates, are used to enhance the stiffness of the thigh.

In this way, the middle 3D printing part was designed and manufactured separately from the thigh lateral plate, which simplifies the difficulty of design, processing, and assembly, reduces the weight and improves the strength of the thigh and the reliability of the system. What is more, to realize the organic integration of hydraulic pipeline and structure, 3D printing technology is used to manufacture key parts, such as the thigh cylinder, which includes knee and hip cylinders, accumulator, multiple hydraulic pipelines and radiator fin, as well as the hip hoseless hydraulic joint (HHJ), which consists of 2 pipelines. Compared with the subtractive manufacturing, the main superiorities of 3D printing are the ability to freely design and manufacture complex structures, as well as rapid prototyping [26].

#### Shank and wheel design

**Fig. 10.**
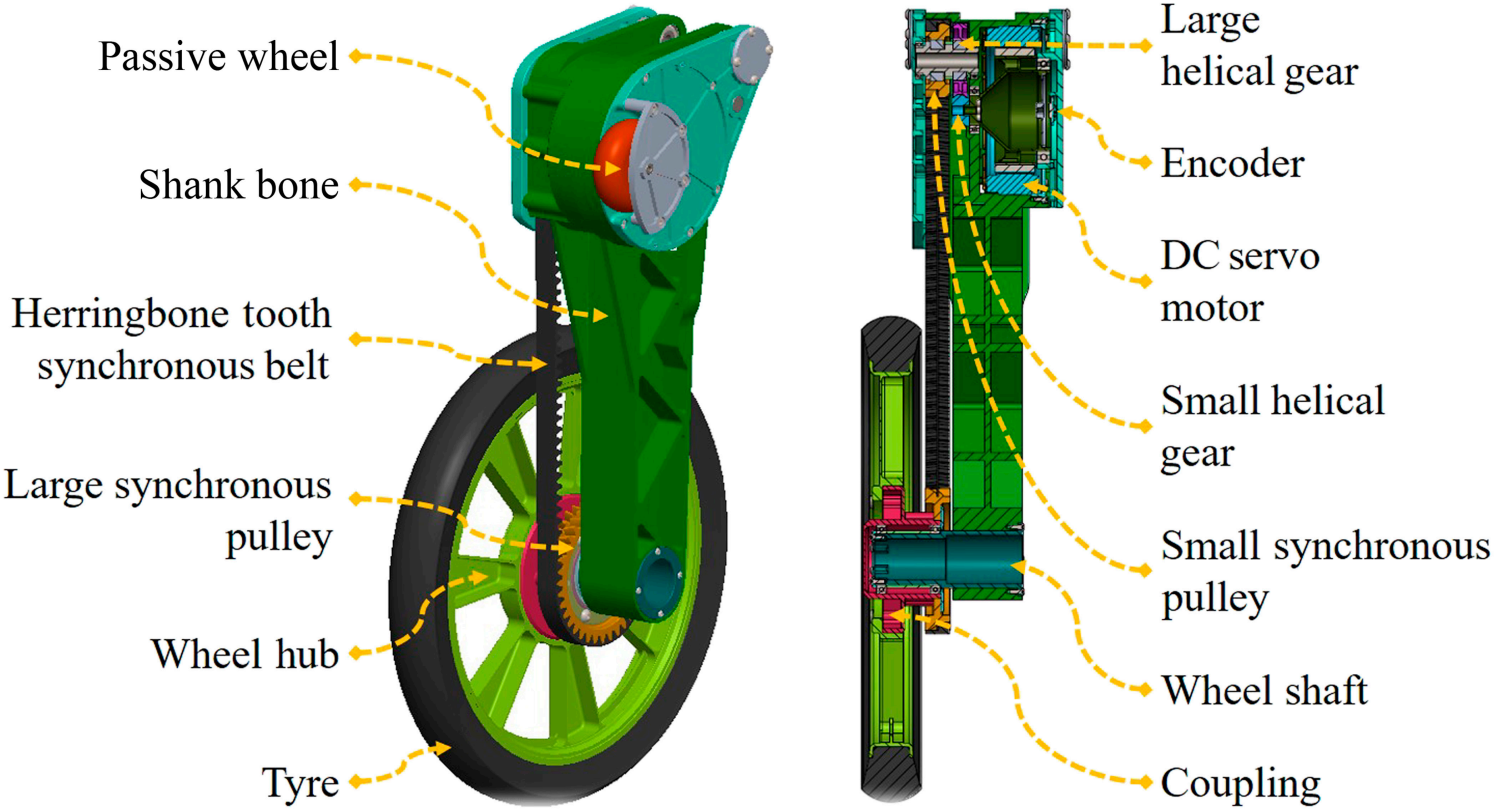
The overview of the calf.

The main factors considered in the shank and wheel design include environmental adaptability, lightweight, and low inertia. Figure 10 shows the structure of the shank and the wheel. The DC brushless servo motor (TQ-ILM115x25), which drives the wheel through the helical gear (reduction ratio: 2.5) and synchronous belt (type: CEY896, reduction ratio: 2) 2-stage reduction transmission, is designed at the calf near the knee joint. A passive wheel is placed at the knee. WLR-3P can move like DRC-HUBO when it kneels on the ground [6]. In such case, the advantage of this design is to reduce the inertia of the shank. Compared with WLR-II’s calf inertia of 136,425 kgmm^2^, WLR-3P’s calf inertia has been reduced by more than half. Since the impact force from the ground directly acts on the wheel, the synchronous belt can protect the motor from the impact and improve the environmental adaptability of WLR-3P.

The general force control based on a dynamic model of the robot can greatly improve motion performance, which depends on the accuracy of the dynamic model. Table 4 lists the mass of each part and the moment of inertia around the rotating shaft of the joint. Most structural parts of WLR-3P are made of aviation aluminum 7075-t6, and 3D printing materials are made of high-strength aluminum alloy. These lightweight materials minimize the weight of WLR-3P.

**Table 4.** Component parameters of WLR-3P.

Name	Mass (kg)	Link length (m)	Inertia(kg·mm^2^)
Wheel	1.7	0.17	25,151
Shank	4.45	0.365	62,380
Thigh	6.1	0.37	66,727
Pelvis	27.34	0.247	486,605
Trunk	27.86	0.199	497,493

### The hydraulic system design

WLR-3P hydraulic system comprises an HPU and the hydraulic power transmission system. The former mainly solved the problem of power autonomy, while the latter mainly solves the problem of power transmission by hoseless design.

#### Hoseless design

**Fig. 11.**
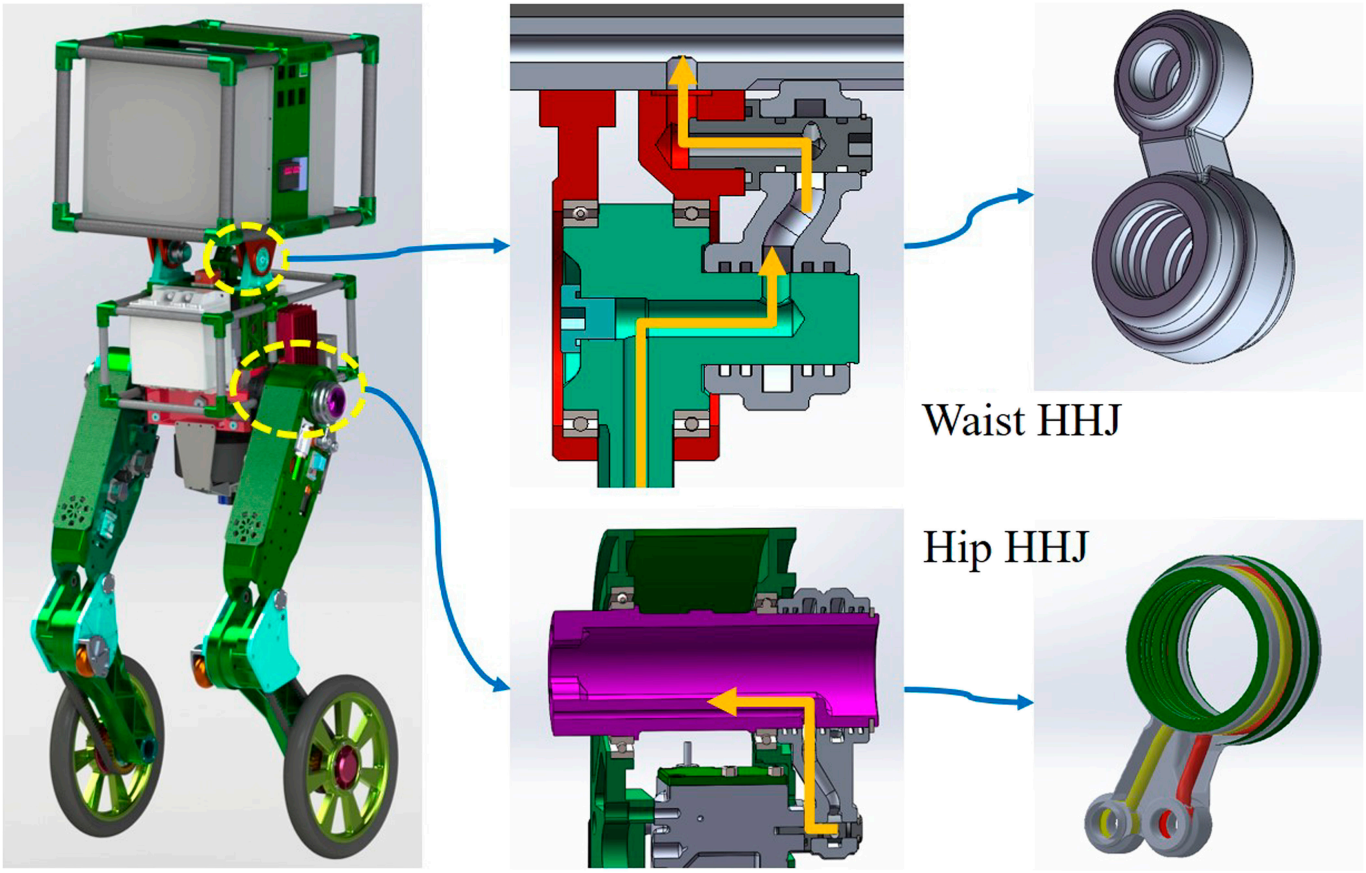
The hip and waist HHJ of WLR-3P.

Hydraulic biped robots, like HIT-Biped [27], HYQ [28], and CB [25], all adopted the rubber hose to connect HDUs with the HPU. Although it is simple and effective, the hose across rotating joints may adversely affect the motion of robots and cause unpredictable disturbances. WLR-3P realizes hoseless design through ingenious hoseless hydraulic joint and cylinder valve skeleton integration [20]. As shown in Fig. 11, hip and waist HHJ parts are all produced by 3D printing. The oil routing part and the bearing part of the joint are designed separately, which avoids the possibility of bias leakage caused by the misalignment of the oil seal groove and the bearing. More importantly, the hollow shaft of hip HHJ allows wires to pass through the hip axis, and the decreased exposure helps to protect the wires from the external harsh environment. The hoseless design can improve the reliability of the robot hydraulic system and increase the flexibility of the joint rotation.

#### HPU design

The miniaturization and integration of a hydraulic power unit (HPU) are the major difficulties in the field of hydraulic robot research, mainly due to the miniaturization of pump and engine as well as system heating. Unlike the quadruped robot [29], WLR-3P does not have a large space for HPU installation. This paper puts forward an innovative design idea to realize the miniaturization and integration of HPU. First, the gear pump is driven by a customized external rotor motor through a micro-coupling. The high-pressure oil from the pump passes through the micro-accumulator and filter to provide the hydraulic power required by the system. Second, aiming at the heating problem of the system, a multifunctional oil tank that integrates heat dissipation, filtration, and pressure stabilization is designed. As a result, the HPU can output hydraulic power with a maximum pressure of 21 MPa and a flow rate of 20 l/min.

**Fig. 12.**
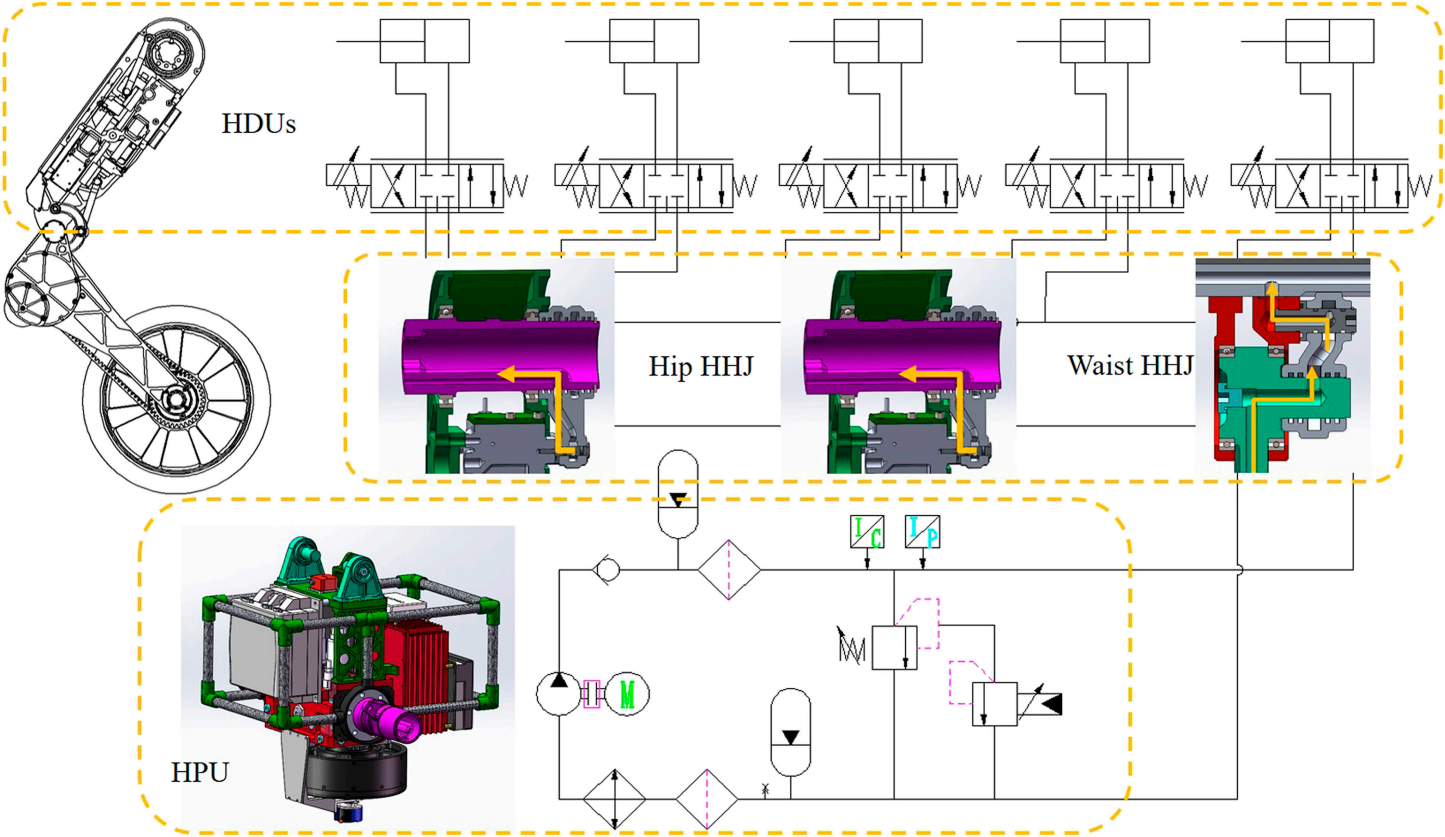
Hydraulic schematic of a complete hydraulic system.

The schematic diagram of WLR-3P hydraulic system is shown in Fig. 12. The motor drives the hydraulic pump to output high-pressure oil, which is directly transmitted to each HDU through a one-way valve and a high-pressure filter. The high-pressure oil pipeline is also equipped with an accumulator to eliminate the pressure pulsation caused by the gear pump. Temperature and pressure sensors detect the pressure and temperature of high-pressure oil, respectively. After passing through the filter and cooler, the return oil from HDUs is directly connected to the oil suction port of the pump to form a closed circuit. The accumulator on the low-pressure oil pipeline acts as the oil tank, compensating the volume difference of hydraulic oil caused by the single-acting hydraulic cylinder and providing a small amount of back pressure to improve the oil absorption performance of the pump. There is also an electromagnetic relief valve between the high-pressure and low-pressure oil pipelines to set the overflow pressure of the system.

**Fig. 13.**
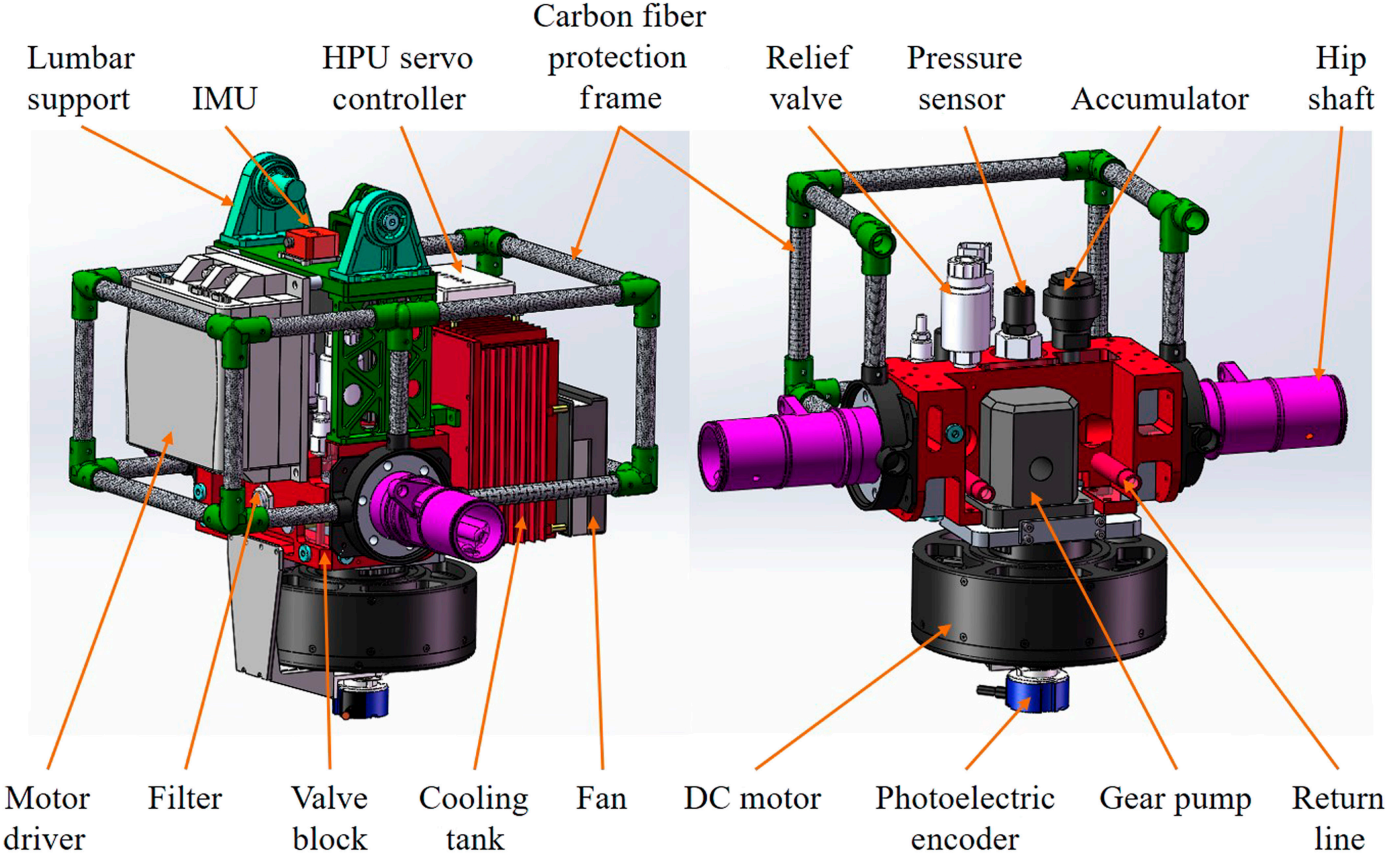
The structure and composition of HPU, which is also the pelvis of WLR-3P.

Figure 13 shows the structure and composition of HPU. The miniaturization and integration of HPU make the robot get rid of the restriction of rubber hose, which makes the robot move more flexibly and freely.

## Control System of WLR-3P

In this section, the robot control system adopting hierarchical and distributed control architecture is proposed. The upper controller is responsible for the motion planning and decision-making of the robot, and the lower controller is responsible for the servo control of each joint. Information is transmitted in real time through CAN bus and EtherCAT bus.

### Electronic system

**Fig. 14.**
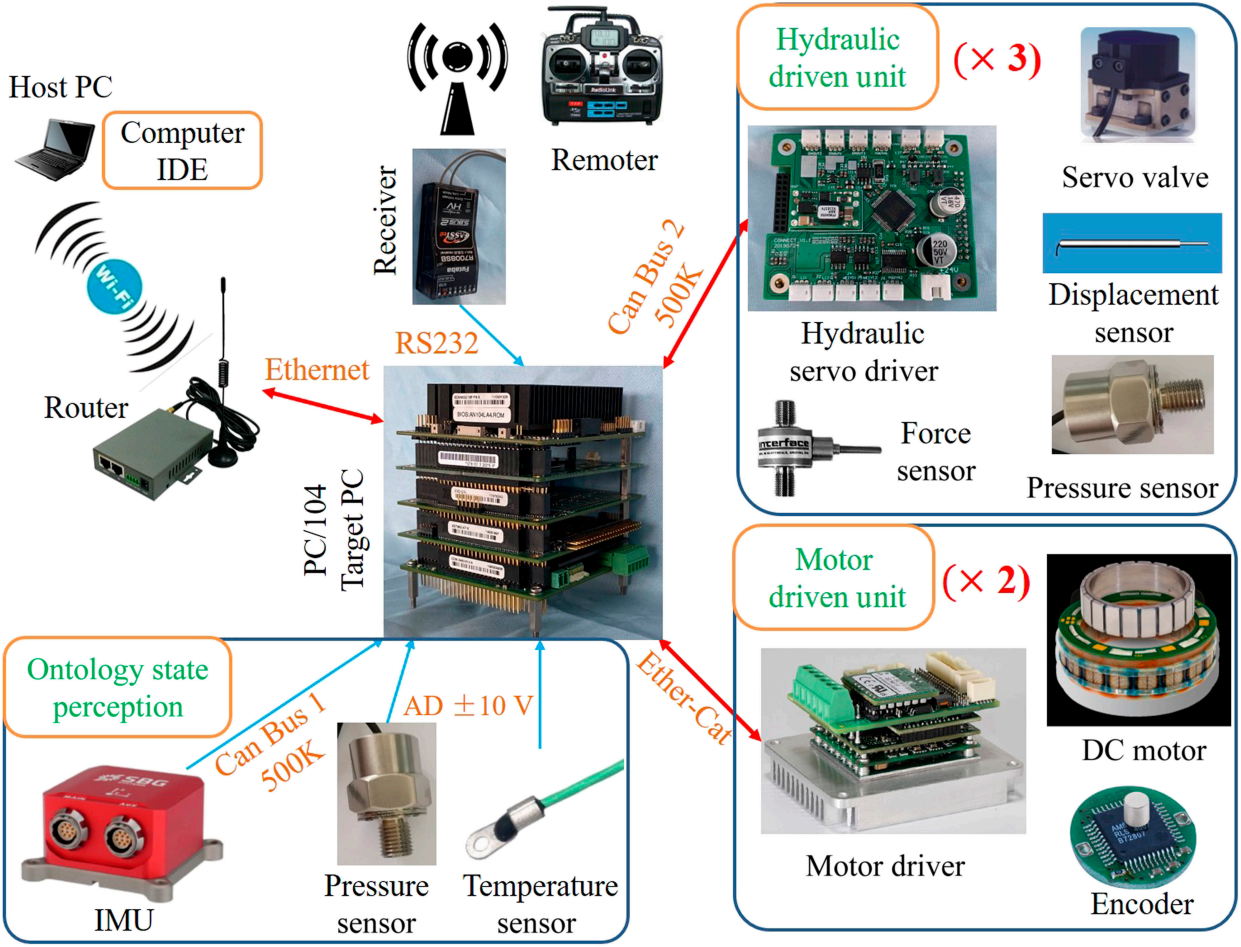
Electronics system of WLR-3P.

The electronic system of WLR-3P is just like its nervous system. It collects all kinds of information from the whole body and converts them into the electrical signal through various sensors. Then, the electric signal is transmitted to the brain (central controller). After calculation and judgment, the brain sends control instructions to each actuator. As shown in Fig. 14, the industrial controller based on PC104 is used as the central controller. The PC104 controller has the advantages of small volume, high reliability, and extensibility. In addition, the central controller equipped with Intel Atom D525 (1.8 GHz, Dual-core) processor is equipped with a 32-bit QNX real-time control system to realize high-speed real-time control over the whole robot. The PC104 controller consists of 2 CAN buses and one EtherCAT bus. Ether-CAT bus communicates with motor drivers (Elmo G-TWI 25/100EE) of driving wheel, and CAN bus 2 communicates with the hydraulic servo drivers, which are used to drive HDU distributed at various joints. The ontology state perception of WLR-3P includes inertial measuring unit (IMU) (Phillipse2-N-G4A3-B2), pressure sensor, and temperature sensor to monitor the oil pressure and temperature information of each part. The human–computer interaction part consists of a PC terminal that communicates via Wi-Fi and a remote controller communicating via radio.

### Control strategy

**Fig. 15.**
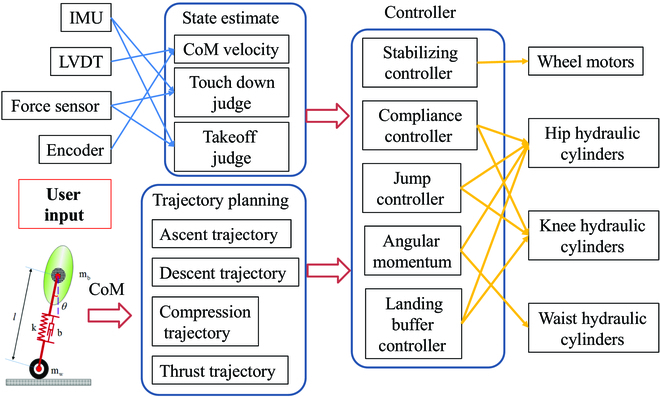
The control frame for WLR-3P.

The wheel-legged robots have high maneuverability and excellent obstacle-crossing performance in unstructured environments, but the stability is poor and the control system is complex due to the large number of degrees of freedom and the narrow support area. This part introduces the control strategy of WLR-3P. WLR-3P’s balance controller adopts the wheel inverted pendulum model and PD control based on linear quadratic regulator driving wheels to realize self-balancing control [30]. Based on the balance controller, the compliance controller is added to deal with complex terrain. The stiffness and damping adjustment of the virtual leg can be realized by adjusting the impedance parameters of hip and knee joints. The 2-mass spring damped inverted pendulum model [31] is applied to the jump control to realize the trajectory planning of takeoff, ascent, descent, and compression. Then, the jumping control of WLR-3P is realized by judging the time of taking off and landing by IMU and force sensor at joints. According to the law of conservation of angular momentum, the swing of a waist joint is adjusted in the process of jumping for attitude control. The control frame of WLR-3P is shown in Fig. 15. The input of WLR-3P control system consists of a state estimator with the information provided by its sensors and a model-based trajectory planner. The control commands are output to the driving wheels and hydraulically driven joints by controller including balance controller, compliance controller, jump controller, angular momentum conservation controller, and landing buffer controller.

## Experimental Results

In this section, we will describe several initial experiments we performed to evaluate the design methods and reliability of the whole system. The hardware performance of WLR-3P is evaluated from 2 aspects: balance performance and explosive force. This result serves as a solid basis for future algorithm testing.

### Balance performance experiment

Fast-moving and squatting are the most basic movements to verify the balance performance of wheel-legged robot. When moving fast, the center of mass (COM) of the robot changes rapidly in the horizontal direction, while COM changes rapidly in the vertical direction when squatting.

**Fig. 16.**
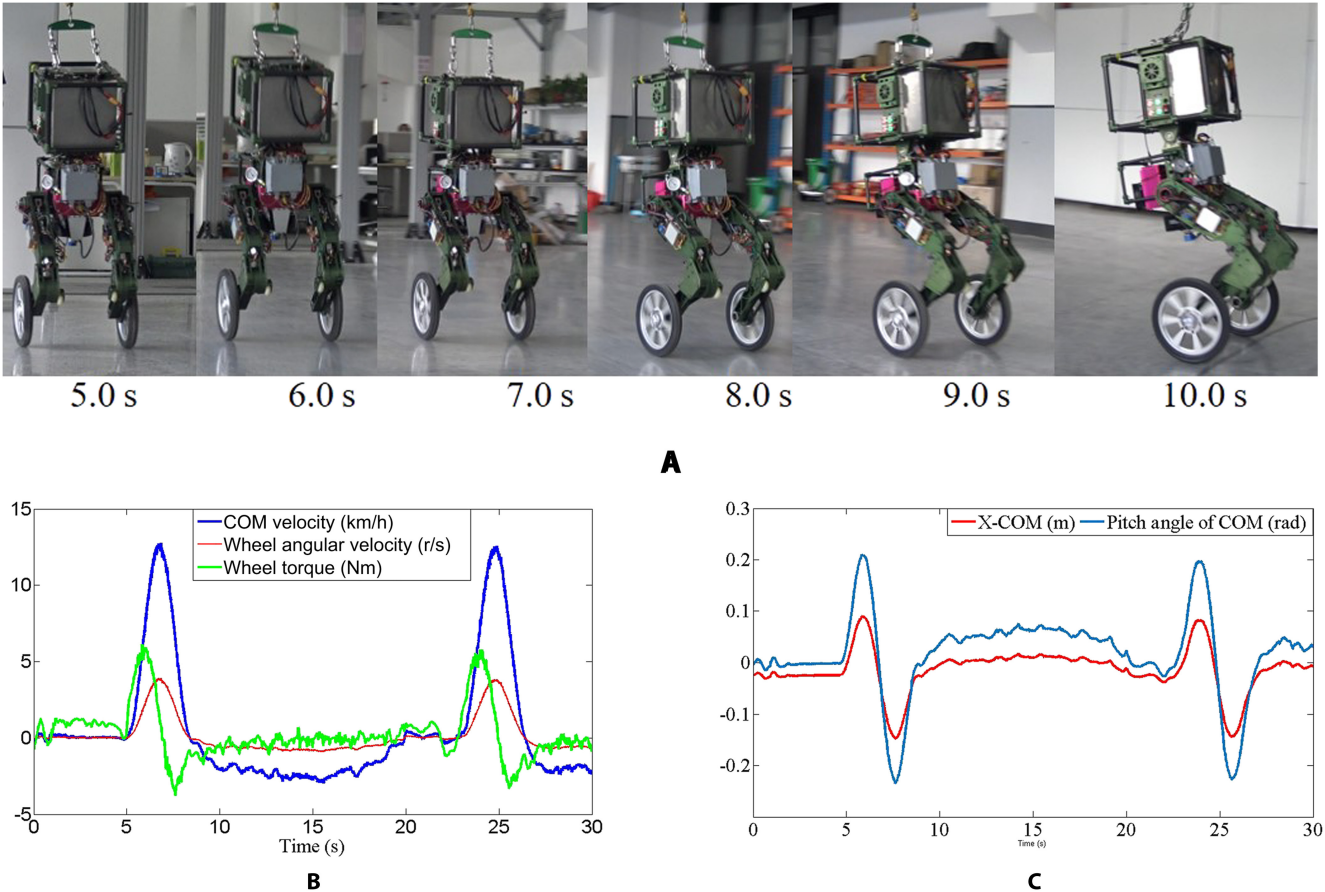
Fast-moving test. The snapshots on the top (A) show the balance ability of the robot when it moves rapidly. The left graph below (B) quantitatively shows COM velocity, wheel angular velocity, and driving torque in the 2 speed tests. The right graph (C) shows the pitch inclination angle of COM and the horizontal distance relative to the center of wheel.

The fast-moving test can verify the maximum speed of the robot on the ground, which also tests the acceleration and deceleration performance and balance ability of the robot. In Fig. 16, WLR-3P leans forward from the static state, accelerates, decelerates, and brakes. WLR-3P returned to the starting point again and repeated the fast-moving test experiment. When the robot moves at high speed, it will cause lateral instability due to mechanical errors and uneven ground. High-frequency adjustment of hydraulic joints is required to improve the lateral stability of the robot.

The experimental results show that the moving velocity of WLR-3P can reach 13.6 km/h on the flat ground, and the angular velocity of the driving wheel is 22 rad/s. Theoretically, the angular velocity of the driving wheel can be higher, but the acceleration distance is limited to 5 m due to the insufficient laboratory area. The rated angular velocity of DC brushless servo motor (TQ-ILM115x25) is up to 1,300 r/min, and the angular velocity of the wheel can reach 27 rad/s after being decelerated by the reducer. When WLR-3P is under the maximum acceleration, the motor just keeps up with the rated torque of 5.4 Nm, far behind the peak torque of 12.2 Nm. When WLR-3P accelerates or decelerates, the horizontal shift of COM (>10 cm) and the inclination angle in the pitch direction (>13°) reflect the balance performance and maneuverability of the robot, while the stability margin of the wheel-legged robot is very narrow, close to a line.

**Fig. 17.**
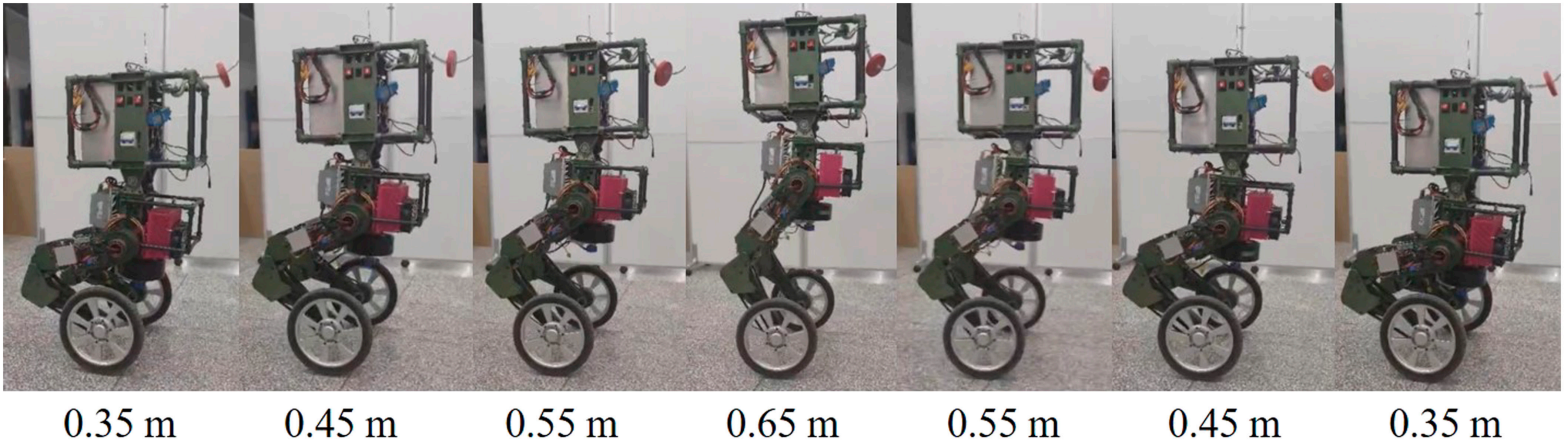
The snapshots from squat that COM is within the height range of 0.35 and 0.65 m.

**Fig. 18.**
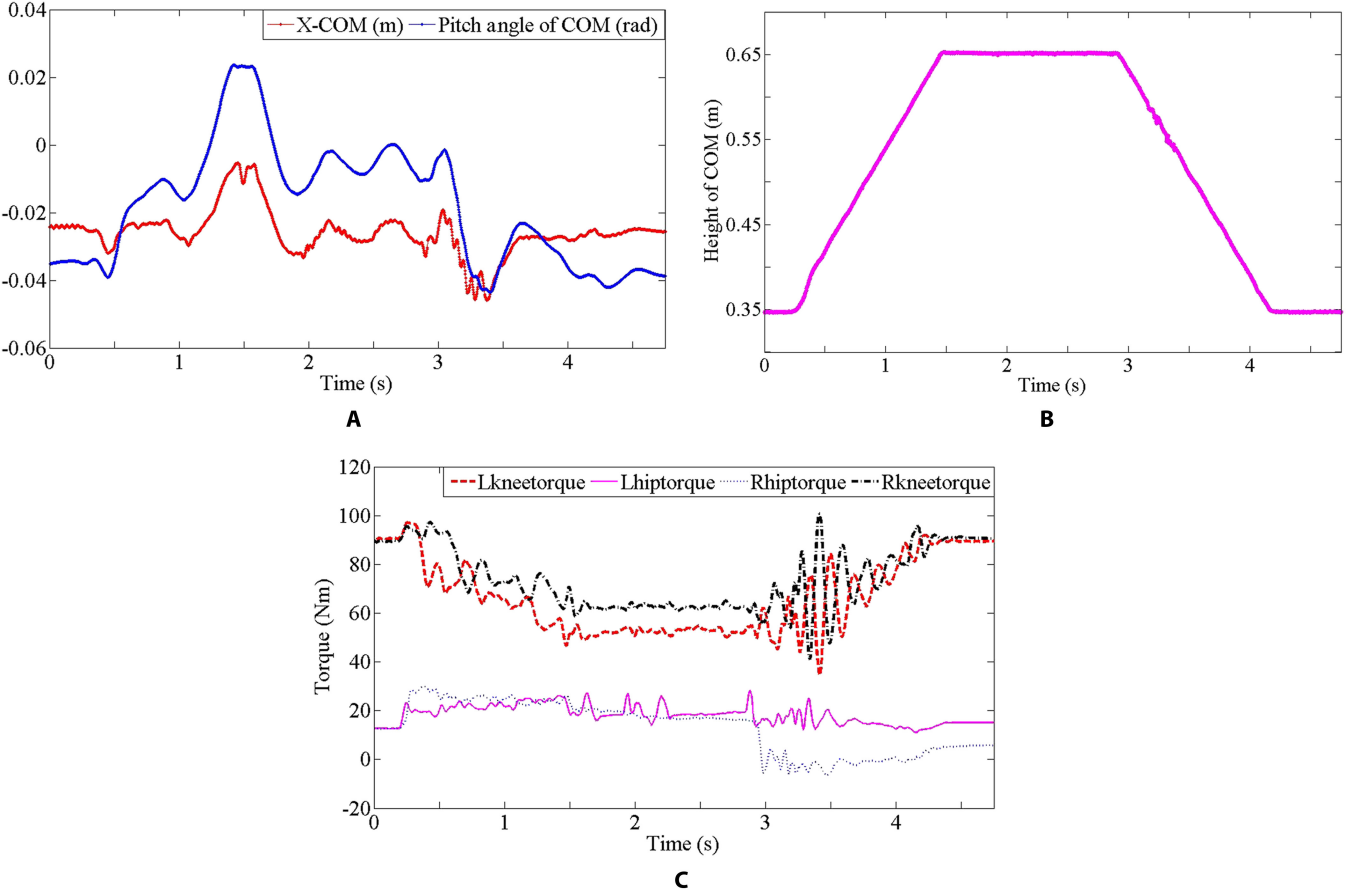
COM adjustment for squat. (A) Offset of COM in the horizontal direction and the inclination angle in the pitch direction. (B) Height change of COM in vertical direction. (C) Knee and hip torque changes in the squat process.

The influence of COM height on the balance is also worth discussing. In Fig. 17, WLR-3P stands up from deep squat and then gradually squats down to return to its original state when its centroid reaches 0.65 m. In the squatting process, COM ascents and descents uniformly in the vertical direction, as shown in Fig. 18B. To achieve balance, COM also needs to be adjusted promptly in the horizontal direction. The adjustment of COM in the horizontal direction and the change of the tilt angle in the pitch direction are shown in the Fig. 18A. Figure 18C shows the joint torques during the squatting process. WLR-3P squatting mainly depends on the knee joint torque, and the higher the centroid, the smaller the torque required. The main function of the hip joints is to adjust the horizontal deviation and inclination angle of COM with the knee joints to maintain balance.

The fast-moving and squatting tests demonstrate the horizontal and vertical balance ability of WLR-3P, which also verifies the hardware system reliability of the wheel-legged robot, laying a solid foundation for the following more complex moving tasks.

### Jumping experiment

**Fig. 19.**
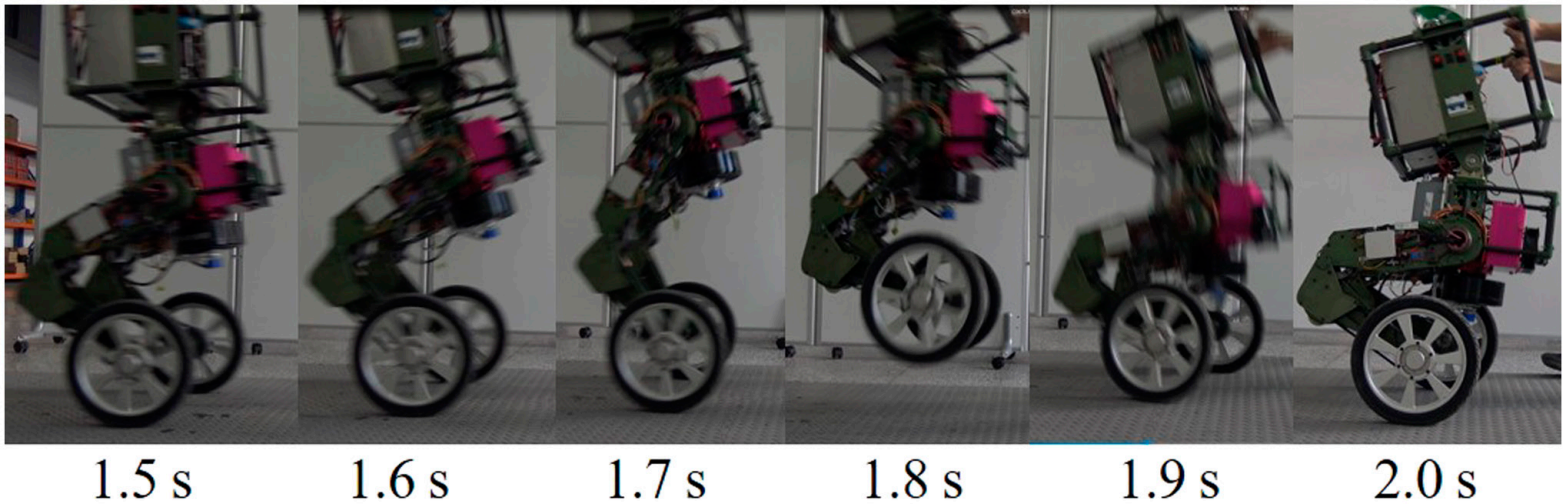
The snapshots of the jump. WLR-3P completed takeoff, flight, and landing process in 0.5 s.

Jumping requires the robot to have a very strong and fast rising force so that the robot can take off in a very short time. At the same time, the HPU provides enough power to ensure that HDUs can obtain enough driving force and speed. These 2 key elements are the prerequisite for the robot to jump. The landing buffer test mainly verifies the impact resistance of the structure. Figure 19 shows the process of the jump within 0.5 s.

**Fig. 20.**
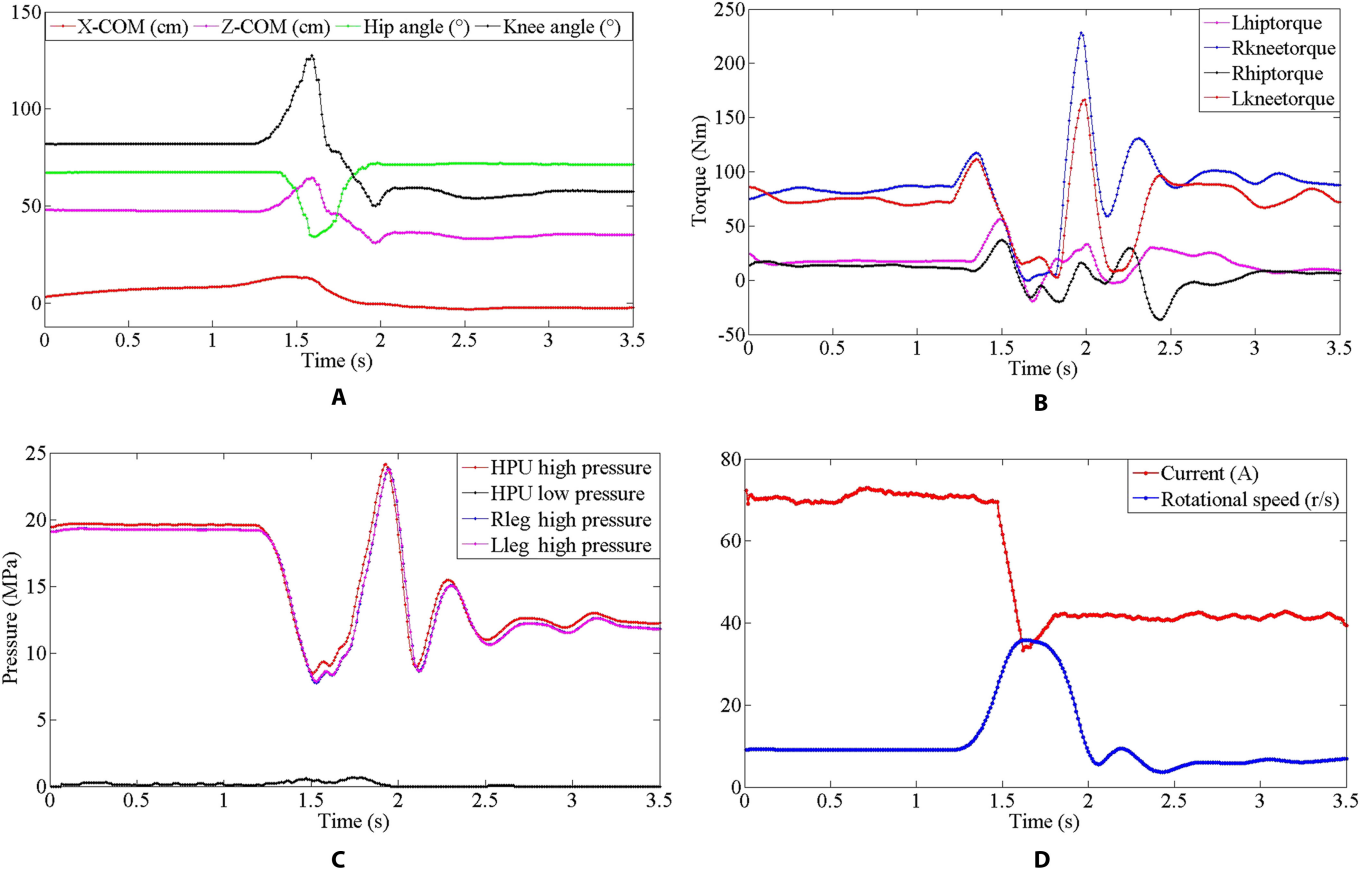
Jump to verification of explosive force and shock resistance. (A) Change of COM in horizontal and vertical directions and angle change of knee and hip joint (the angle changes of 2 legs are the same). (B) Torque changes of knee and hip joint. (C) High and low pressure of HPU and high pressure of the 2 legs. (D) Current and rotational speed variation of HPU motor.

As shown in Fig. 20A, when the robot jumps, the knee joint angle extends from 82° to 128° within 0.3 s and then flexed to 50°. The height of COM ascended from 47 to 65 cm and then compressed to 31 cm after landing. To maintain the balance of the robot, COM is also adjusted in real time. Figure 20B shows the changing trend of the torque of knee and hip joints. The knee joint torque changes most obviously. During the jump, there is a small fluctuation in the hip joint. The biggest fluctuation of hip torque is in the takeoff stage, and the change is small when landing. The knee joint mainly bears the impact of landing. As can be seen from Fig. 20B, there is a large gap between the maximum driving torque of knee joint (117 Nm) and the maximum theoretical design value (242 Nm). In the landing phase, the knee joint torque rapidly rises to 228 Nm due to the strong impact, which is within the impact range that the structure and drive unit can withstand. The reason for insufficient driving force during takeoff and excessive impact force during landing is that the response speed of the HPU is lower than that of HDU. It can be seen from Fig. 20C and D that although the motor speed of HPU increases to compensate the hydraulic power flow of the system after takeoff, the response speed of the HPU is still lower than that of HDU, so the pressure drop of the system cannot be prevented. Therefore, how to compensate the flow output of HPU to maintain the pressure during the jump process is an urgent problem to be solved in the follow-up of this work. In this paper, the reliability of the hardware system is verified by the preliminary jumping experiment.

The successful jumping of the WLR-3P robot has verified the explosive force, high power density, and fast response ability of the hydraulic drive system. Frequent jumping and landing impact also fully test the high strength and impact resistance of the structure. Summarizing the experimental experience, it can be found that the lighter the weight of the robot and the stronger the explosive ability of the drive system, the higher the robot can jump. In particular, the smaller the proportion of the weight of the legs to the total body weight, the higher the instantaneous output force and speed of the knee joints, which will have a more significant impact on the jumping performance of the robot. In the process of developing the WLR-3P robot, the above factors are fully considered, and the design requirements and implementations proposed in this paper meet the high mobility requirements of the robot.

## Conclusions

This paper introduces the development process of a highly integrated hydraulic wheel-legged robot. First, the design method for WLR-3P for dynamic motion and power autonomy is presented. The design process aims to substantially improve the mobility and environmental adaptability of wheel-legged robot. After that, the robot control system adopting hierarchical and distributed control architecture is introduced. Finally, experimental tests were conducted on the system for fast-moving, squatting, and jumping. The balance control ability is verified by the fast-moving and squatting experiments, while the reliability of the system is verified by the jumping experiment. However, there is still a gap between the actual performance and the theoretical design performance, which gives clear direction for future work. That is, how to match the output power of the HPU and the power required by HDU. For the demands on driving power and robot motion, it is necessary to consider the engineering problems such as heat dissipation and noise. What is more, the miniaturization and customization of hydraulic components is also one of the future research directions. This work has provided a good foundation for further work toward the development and control of a wheel-legged robot. More importantly, this research verifies the design and control strategy by implementing them in real hardware system.
